# Zinc Oxide Films with High Transparency and Crystallinity Prepared by a Low Temperature Spatial Atomic Layer Deposition Process

**DOI:** 10.3390/nano10030459

**Published:** 2020-03-04

**Authors:** Ming-Jie Zhao, Zhi-Tao Sun, Chia-Hsun Hsu, Pao-Hsun Huang, Xiao-Ying Zhang, Wan-Yu Wu, Peng Gao, Yu Qiu, Shui-Yang Lien, Wen-Zhang Zhu

**Affiliations:** 1 School of Opto-electronic and Communication Engineering, Xiamen University of Technology, Xiamen 361024, China; 2015000077@xmut.edu.cn (M.-J.Z.); 18359225079@163.com (Z.-T.S.); xyzhang@xmut.edu.cn (X.-Y.Z.); wzzhu@xmut.edu.cn (W.-Z.Z.); 2 Fujian Key Laboratory of Optoelectronic Technology and Devices, Xiamen University of Technology, Xiamen 361024, China; 3 School of Information Engineering, Jimei University, 183 Yinjiang Road, Jimei, Xiamen, Fujian 361021, China; ph.huang@jmu.edu.cn; 4 Department of Materials Science and Engineering, Da-Yeh University, Changhua 51591, Taiwan; wywu@mail.dyu.edu.tw; 5 Xiamen Institute of Rare Earth Materials Research, Chinese Academy of Science, Xiamen, Fujian 361024, China; peng.gao@fjirsm.ac.cn; 6 Key Laboratory of Green Perovskites Application of Fujian Province Universities, Fujian Jiangxia University, Fuzhou 350108, China; yqiu78@hotmail.com

**Keywords:** zinc oxide, spatial atomic layer deposition, low temperature, thin films, crystallinity

## Abstract

Zinc oxide (ZnO) attracts much attention owing to its remarkable electrical and optical properties for applications in optoelectronics. In this study, ZnO thin films were prepared by spatial atomic layer deposition with diethylzinc and water as precursors. The substrate temperature was varied from 55 to 135 °C to investigate the effects on the optical, electrical, and structural properties of the films. All ZnO samples exhibit an average transmittance in visible and near-infrared light range exceeding 80% and a resistivity in the range of (3.2–9.0) × 10^−3^ Ω·cm when deposited on a borosilicate glass with a refractive index of ≈1.52. The transmittance, band gap, refractive index, and extinction coefficient are rarely affected, while the resistivity only slightly decreases with increasing temperature. This technique provides a wide process window for depositing ZnO thin films. The results revealed that the films deposited at a substrate of 55 °C were highly crystalline with a preferential (1 0 0) orientation. In addition, the grains grow larger as the substrate temperature increases. The electrical properties and reliability of ZnO/PET samples are also studied in this paper.

## 1. Introduction

Zinc oxide (ZnO) attracts much attention owing to its remarkable electrical and optical properties for applications in optoelectronic devices, such as solar cells, gas sensors, light-emitting diodes (LEDs), and thin-film transistors (TFTs) [1,2,3,4]. ZnO thin films can be obtained by a wide variety of growth methods, including chemical vapor deposition (CVD), pulsed laser deposition (PLD), molecular beam epitaxy (MBE), sputtering, solution-based methods, and atomic layer deposition (ALD) [5,6,7,8,9,10]. Among these methods, ALD has been considered as a promising method for depositing high-quality, thin films due to its precise control of film growth, low surface damage, good conformal coverage over 3D substrates, and excellent uniformity on a large-area substrate. However, conventional ALD is not always suitable for mass manufacturing due to its low deposition rate, which is strongly limited by the long purge time needed to remove excess precursors and reactants. Recently, this drawback has been overcome by the development of the spatial ALD (sALD) technique, in which a moving substrate is sequentially exposed to precursors dosed by spatially separated multiple spray heads, rather than dosed temporally by a single head, so that the long purge time can be eliminated [10,11,12]. Therefore, sALD combines the advantages of conventional ALD with a high deposition rate. Besides, the sALD process can be operated under atmospheric pressure, which makes it more cost-effective than other vacuum-based film deposition methods. With these advantages, sALD is becoming an emerging technique for the growth of high-quality thin films in industry. 

In recent years, the emergence of flexible electronics raises a new requirement for the manufacturing of optoelectronic devices [13,14,15,16,17,18,19]. The devices need to be fabricated at a low temperature to be compatible with thermally sensitive, flexible substrates. Some efforts have been made to apply an ALD or sALD-based process to deposit ZnO films and fabricate a related device on a plastic flexible substrate, such as polyimide (PI) or polyethylene naphthalate (PEN), whose highest endure temperatures are ≈300 and ≈175 °C respectively [15,17]. However, the typical deposition temperature of the sALD procedure to obtain ZnO films from diethylzinc (DEZ) and water (H_2_O) is in the range of 150–250 °C, which is higher than that for conventional ALD (120–180 °C) [12,17,20,21,22]. Additionally, the thin film properties are strongly dependent on the deposition or post-annealing temperature. Thus, the films usually need to be deposited or post-annealed at a higher temperature to achieve the optimal performance. These processes are only applicable to PI and PEN substrates. However, these substrates are either poor in optical properties or expensive. Ploy(ethyleneterephthalate) (PET) is a cost-effective flexible substrate with good optical properties, but the application of ZnO sALD process to a PET substrate is limited, since it can only endure a temperature of <120 °C. The deposition temperature can be reduced to below 100 °C by using a stronger oxidant, such as ozone (O_3_), as the precursor. However, ozone is easily decomposed, so it needs to be prepared instantly by an expensive facility. Another possibility to deposit ZnO at low temperatures is plasma enhanced ALD (PEALD), in which an in-situ O_2_ plasma treatment is added after each ALD cycle involving the standard DEZ and H_2_O sequence to enhance the ALD process and hence lower the deposition temperature [23]. However, PEALD suffers from the same problems as conventional ALD, such as the low deposition rate and the costliness of a vacuum-based facility; hence, it is less attractive for mass manufacturing due to the low throughput and massive investment. Therefore, developing a low-temperature sALD process using DEZ and water (H_2_O) precursors is still promising for the application in flexible electronics in the current stage.

In this work, we prepare ZnO films with high transparency and crystallinity at a wide temperature range by an sALD technique. The lowest process temperature can be as low as 75 °C. Therefore, it can be applied to a PET substrate. ZnO thin films have been successfully deposited on a PET substrate by this technique and show excellent reliability after the bending test. 

## 2. Materials and Methods 

**Figure 1:**
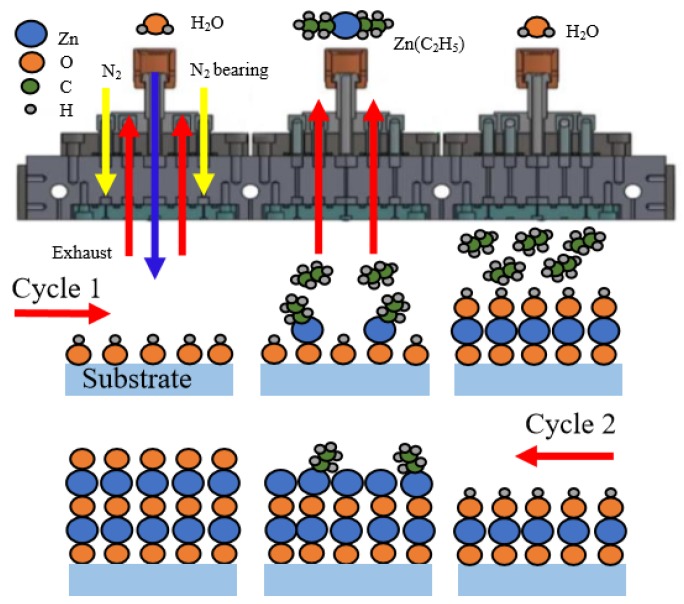
Schematic description of the deposition mechanism of the sALD-ZnO thin film.

ZnO films were deposited on borosilicate glass (50 mm × 50 mm), silicon wafer (4-inch diameter), or PET (50 mm × 50 mm) by a home-built spatial ALD system that was equipped with multiple spray heads for precursors, inert gas, and the exhaust, as shown in Figure 1. DEZ and deionized water vapor (H_2_O) were used as precursors due to their high vapor pressures, strong reactivities, and broad ranges of deposition temperature [22]. Nitrogen gas (N_2_, 99.99% in purity) was used as the carrier gas for both precursors and as the dilute gas in the reactor. The DEZ and H_2_O precursor containers were kept at 27 °C by thermostatic water baths to provide stable vapor pressures of the precursors. The gas delivery pipelines were maintained at 37 °C to prevent the precursors from condensation. Each head for precursors was flanked by two exhaust heads to remove the redundant vapors and any by-products. The substrate was placed at a distance of 0.3 mm from the spray heads. The squared substrate holder with a side length of 156 mm, which was the upper limit of the dimension of the substrate, moved forward and backward horizontally at a steady speed of 15 cm/s. The maximum moving distance of the substrate holder is 600 mm and it spends 4 s completing one ALD cycle. The substrate was exposed to one DEZ head and two H_2_O heads for one ALD cycle. The films were grown by repeating the ALD cycles. A total of 1000 cycles was executed for each sample deposited at different temperatures when evaluating the deposition rates. The average film thicknesses of ≈90 nm was obtained from nine points distributed on the glass substrate. The deposition rates were defined as the quotient of the average thickness to the number of cycles. The reason for having such film thickness is to reduce the error of optical measurements and to make it easy to observe structural changes in the film. The substrate temperature was varied from 55 to 135 °C for different ZnO films. The deposition parameters are summarized in Table 1.

The film thicknesses (*t*) were measured by a step profiler (KLA-Tencor D-500) and verified by an ellipsometer (J. A. Woollam Co., Inc., Lincoln, NE, USA, M-2000). The transmittance and reflectance spectra of the films were measured by a spectrometer (Hong-Ming Technology, Taiwan, MFS-630). A borosilicate glass substrate with a refractive index of ≈1.52 was used for these measurements. The refractive index (*n*) and extinction coefficient (*k*) were obtained by a spectroscopic ellipsometric measurement using a wavelength in the range of 350–950 nm. In the measurement, a silicon wafer was used as the substrate; the air roughness model, i.e., the “air, air/ZnO, ZnO, silicon substrate-air” four-layer structure, in which the ZnO layer was fitted by a Tauc-Lorentz model, was used for evaluating the *n* and *k* values of the ZnO film. The resistivity, carrier concentration (*N*_c_), and mobility (*μ*) values of the films were detected by Hall effect tests (Side Semiconducter Technology, HMS5000). The structural characteristics of the thin films were investigated using Grazing-Incidence X-ray diffraction (GIXRD, RigakuTTRAXIII, Ibaraki, Japan) spectra [23,24,25]. This technique is usually applied to research thin films, since the penetration of the X-ray is significantly reduced to 1–10 nm. Thus, a higher signal-to-noise ratio can be achieved for the XRD pattern. The surface morphologies of the thin films were observed by field emission scanning electron microscopy (FESEM) (Zeiss sigma 500).

The ZnO film was deposited on a PET substrate by sALD at 75 °C to demonstrate the feasibility of applying on the thermal sensitive, flexible substrate. A bending test was carried out to examine the reliability of the ZnO/PET sample [16,17,18,19]. In the bending test, the ZnO/PET sample was repeatedly bent upward and downward alternatively with a curvature radius of 10 mm. The resistivity was periodically probed between cycles to monitor the change of electrical properties.

## 3. Results

### 3.1. Deposition Mechanism of ZnO Films by sALD

Figure 1 shows a schematic description of the deposition mechanism of the sALD-ZnO thin film. The reaction can be described by the following formulas.

*S*-OH + Zn(C_2_H_5_)_2_→*S*-O-Zn-C_2_H_5_ + C_2_H_6_↑, (1)

*S*-O-Zn-C_2_H_5_ + H_2_O→*S*-O-Zn-OH + C_2_H_6_↑, (2)

Zn(C_2_H_5_)_2_ + H_2_O→ZnO + 2C_2_H_6_↑, (3)

where *S* represents the substrate surface. The symbol ↑ denotes the volatile gaseous phase. In the first half-reaction, hydroxyl ligands are adsorbed on the substrate surface and react with DEZ to form Zn-O bonds and produce volatile C_2_H_6_ gas, as described in Equation (1). In the second half-reaction, H_2_O molecules are adsorbed to the substrate and react with C_2_H_5_ ligands to produce new hydroxyl ligands, as described in Equation (2). Equation (3) described the overall reaction of a complete ALD cycle.

**Figure 2:**
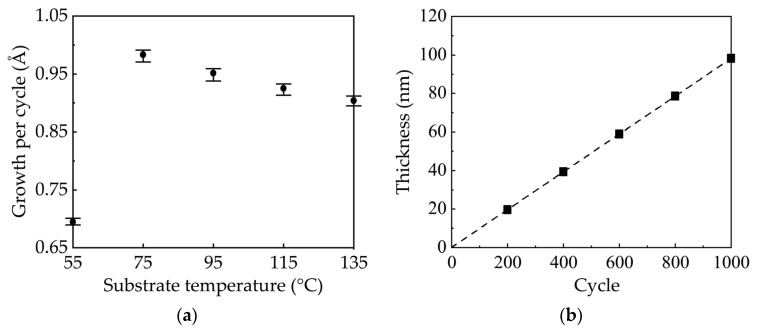
(**a**) Variation in the growth per cycle (GPC) of sALD-ZnO films as a function of substrate temperatures. (**b**) The film thickness as a function of the number of sALD cycles at a substrate temperature of 75 °C.

Figure 2a shows the growth per cycle (GPC) values of ZnO films on borosilicate glass substrate as a function of deposition temperature. The GPC is defined as the ratio of film thickness to cycle number. The average GPC values and error bars were obtained from nine-point film thickness distribution. For all the samples the error bars are less than 1.4%, indicating good uniformity. The GPC first increases and then slightly decreases with increasing temperature. Therefore, the films deposited at different temperatures have different film thicknesses ranging from 79 to 91 nm when executed for the same 1000 cycles. At low temperature, the film growth is dominated by kinetically limited surface reactions due to the insufficient activation of the precursors [22]. Thus, the GPC increases with temperature. At higher temperature, the film growth is limited by the precursor supply and a stable ALD process window is reached [22]. Therefore, the GPC becomes saturated and less dependent on temperature. The slight decrease in GPC is probably due to the desorption of reactive species from the surface as the temperature increases [26]. This variation trend is accordant with previously reported works [20,21,22]. Notably, the lower limit of the ALD process window is only 75 °C, which is significantly lower than those reported in other works [11,12,13,14,20,21,22,27]. The reported temperature ranges for the ALD window show a lot of inconsistencies in the literature. They can be influenced by the use of different precursors, the reactor design, and the pulse/purge time [27,28]. In our case, the reaction of DEZ with H_2_O is exothermic, which is favorable for low temperature deposition. Besides, the moving speed of the substrate holder is 15 cm/s, corresponding to an exposure time of about 267 ms that is similar to that in other works [12,27]. The self-designed sALD system may also play an important role in lowering the process temperature. Although the reason for such a low temperature of the sALD window cannot be accurately assigned, we successfully demonstrate that the lower limit of ALD window can be as low as 75 °C. Such a low temperature enables us to deposit the ZnO films on temperature-sensitive, flexible substrates, including PET. Figure 2b shows the film thickness as a function of the number of ALD cycles at a substrate temperature of 75 °C. It can be seen that the film thickness increases linearly with the number of ALD cycles, which indicates the linear film growth expected for an ALD process [21]. The standard deviation is less than 1%, indicating a good controllability of the film thickness. Furthermore, the deposition rate reaches a high value of 1.48 nm/min at 75 °C, which is several times higher than that of conventional ALD process (typically <0.01 nm/s), and is therefore of special interest for industrial applications due to the possible high throughput [10,20].

### 3.2. Optical Properties of ZnO Films

**Figure 3:**
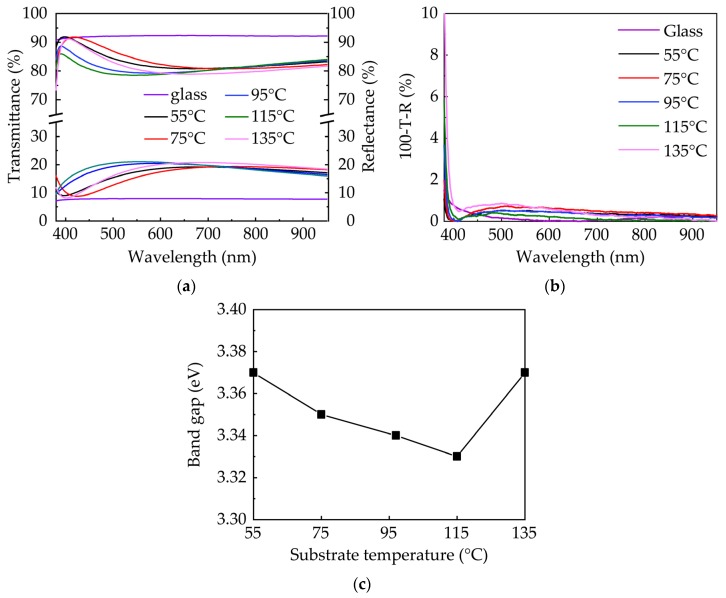
(**a**) Transmittance spectra and reflectance spectra; (**b**) optical losses; and (**c**) optical band gaps (*E_g_*) of sALD-ZnO films with varying substrate temperatures.

Figure 3a shows the transmittance and reflectance spectra of ZnO films deposited on the glass substrate at various substrate temperatures. Since the GPC depends on the substrate temperature, the film thickness varies for different samples with the same number of sALD cycles. The samples all exhibit a high transmittance in visible and near-infrared light range (380–950 nm). Only a small variation of optical properties with substrate temperature was observed. Notice that the transmittance is inversely related to the reflectance. The variation in transmittance should be ascribed to the variation in reflectance primarily, rather than the change in film properties. The variation in reflectance results from the change in interface interference, since the film thickness varies from 79 to 91 nm in this study. To investigate the effect of the substrate temperature on the transmittance of the ZnO thin film, the optical losses in the range of 380–950 nm was calculated as expressed by 100%-*T*-*R* (Figure 3b), where *T* and *R* are the transmittance and reflectance, respectively. The optical losses of all samples are very low (<1%) in the long wavelength range of 400–950 nm regardless of the substrate temperature. However, they sharply increase in the lower wavelength range of 380–400 nm due to the band to band absorption. In addition, the optical losses at the lower wavelength range seem to increase with the substrate temperature, which may result from the light absorption and scattering due to the change in micro-structure indicated by the XRD and FESEM results. However, the increase in optical losses has little effect on the average transmittance. The band gap (*E*_g_) of the ZnO thin film was also extracted by Tauc plot [29] with the equation:

(4) $${\left(\alpha \mathrm{hv}\right)}^{2}=A\left(\mathrm{hv}-E_{g}\right)$$

where α is the absorption coefficient, hν is the energy of the incident light, A is a constant, and *E*_g_ is the band gap. The absorption coefficients of the films were calculated from the transmittance and reflectance spectra using the following the equation:

(5) $$\alpha =\frac{1}{t}\ln\left(\frac{1-R}{T}\right)$$

where *t* is the film thickness, and *R* and *T* are the reflectance and transmittance, respectively. The results are shown in Figure 3c. A band gap of 3.3–3.4 eV is in good agreement with those values reported in the literature [20,22]. The variations in band gap with temperature are negligible, indicating that they are rarely affected by deposition temperature. This is in good accordance with the analysis in the transmittance and reflectance spectra.

**Figure 4:**
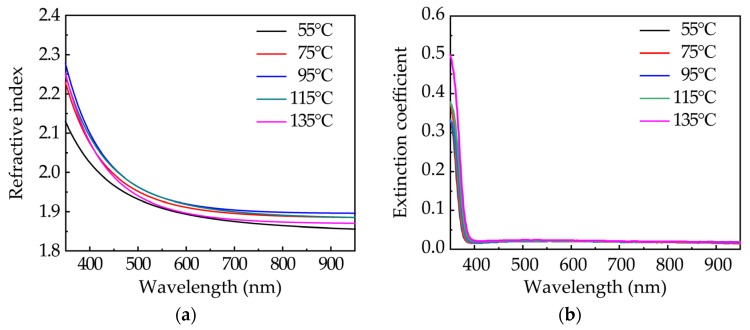
The wavelength-dependent (**a**) refractive indexes and (**b**) extinction coefficients of sALD-ZnO films deposited at various substrate temperatures.

Figure 4 shows the wavelength-dependent refractive indexes (*n*) and extinction coefficients (*k*) of sALD-ZnO films. Silicon wafers were used as the substrate. The refractive index of ZnO film deposited at 55 °C is significantly lower than those of the film deposited at a higher substrate temperature, especially at a short wavelength range. The variation in the refractive index should reflect the change in film density, as they have strong correlations [30,31,32]. It is intelligible that the films deposited at a low substrate temperature have a low film density due to the incomplete reaction. However, the substrate temperature has little effect on the refractive index when the film growth reaches the steady-state ALD phase. The value of around 1.9 at 630 nm is consistent with that reported in the literature [20,21,32]. The *k* values are very similar for all samples. The absorption edge of the *k* spectra was found to be at the wavelength of 375–390 nm, corresponding to the light energy of 3.2–3.3 eV, which is close to the band gap of the ZnO film extracted by Tauc plot. Overall, the optical properties, such as transmittance, band gap, refractive index, and extinction coefficient, are rarely affected by the substrate temperature. This means a wide process window when concerning these optical properties.

### 3.3. Electrical Properties of ZnO Films

**Figure 5:**
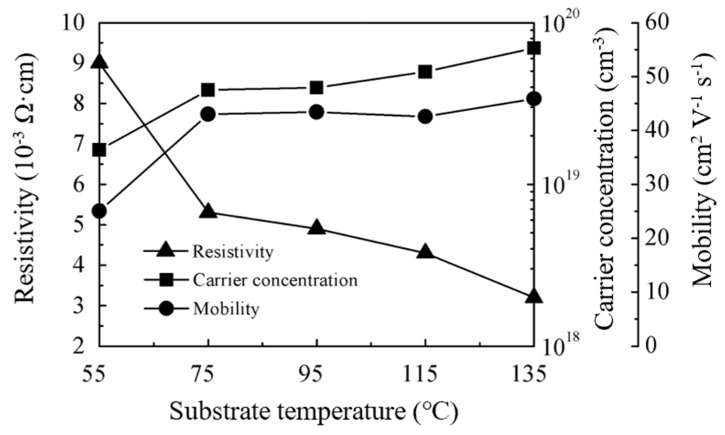
The variations of resistivity, carrier concentration (*N*_c_), and mobility (*μ*) for sALD-ZnO films with deposition temperatures.

Figure 5 shows the resistivity, carrier concentration (*N*_c_), and mobility (*μ*) of ZnO films on a glass substrate. All films exhibit an n-type conductivity and a low resistivity in the range of (3.2–9.0) × 10^−3^ Ω·cm. The resistivity decreases with increasing substrate temperature due to the increase in carrier concentration and mobility. Specifically, the carrier concentration continuously increases from 1.6 × 10^19^ to 7.0 × 10^19^ cm^−3^ as the substrate temperature increases from 55 to 135 °C. The mobility showed a considerable increase from 25 to 43.0 cm^2^V^−1^s^−1^ when the substrate temperature increased from 55 to 75 °C, but changed very little at 75–135 °C. The massive increase in mobility is related to the transition in the growth phase and the orientation transformation in crystallites. A loose packing film is expected when deposited at 55 °C due to the insufficient reaction of precursors. The film becomes denser when the film growth enters the steady-state ALD phase at higher deposition temperature, which contributes to the increase in mobility. This is further supported by the increase in the refractive index. The orientation transformation in crystallites and its contribution to the electrical properties are discussed in the next sub-section.

Regardless of the effects of substrate temperature on the electrical properties, the resistivity in the range of (3.2–9.0) × 10^−3^ Ω·cm is low for an intrinsic ZnO film. Even deposited at 55 °C, the film resistivity is still acceptable. However, the low deposition temperature will benefit its application in flexible electronics.

### 3.4. Structural and Morphological Study of ZnO Films

**Figure 6:**
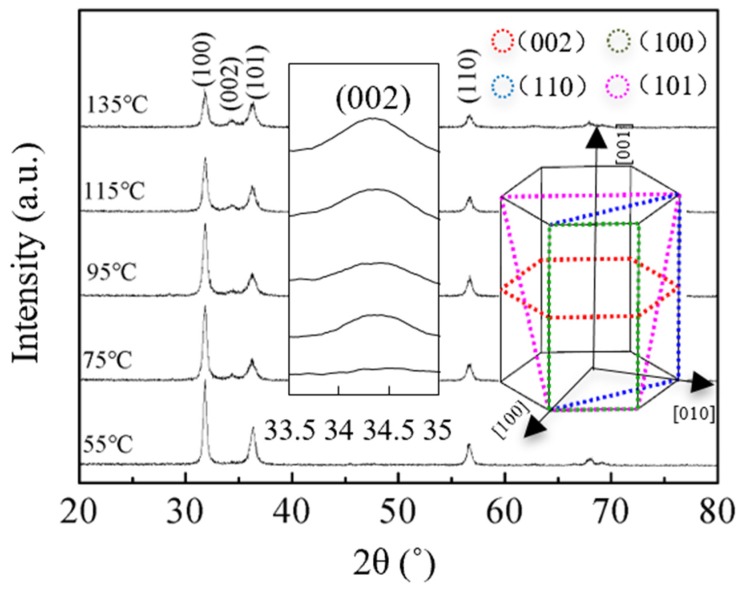
Grazing-incidence X-ray diffraction patterns of sALD-ZnO films deposited at different temperatures.

The structural and morphological characteristics were investigated to understand the electrical properties of ZnO films further. Figure 6 shows the GIXRD patterns of ZnO films on silicon wafer. The existence of multiple diffraction peaks indicates that the films are polycrystalline. These peaks correspond to the wurtzite structure of ZnO [15,21,27]. The crystal structure of hexagonal ZnO wurtzite is shown in the inset of Figure 6. It was reported that (1 0 0) has higher surface energy than (1 1 0) and (0 0 2) [33]. The sALD-ZnO films have the strongest intensity of (1 0 0) peak, which should be ascribed to the low deposition temperature and short expose/purge time (contributes to the high deposition rate). As a result, the adatoms do not have enough time and energy to migrate to the energetically favorable sites. As the substrate temperature increases, the intensity of already existing peaks decreases. Meanwhile, a weak (0 0 2) peak starts to appear at 75 °C. An enlarged plot of (0 0 2) peak is inserted in the inset of Figure 6. It was observed that the intensity of (0 0 2) of the sALD-ZnO film increases with substrate temperature due to the lowest surface energy of it. This result indicates the crystal grows along the c axis of wurtzite structure due to (0 0 2) being the closest packing and energetically favorable plane in a wurtzite structure [34]. Therefore, the adatoms gain more energy at a higher temperature to overcome the energy barrier to get to the energetically favorable sites. The driving force for crystal development comes from the reduction in the Gibbs free energy of the system and the increase of adatom mobility. As a result, the resistivity and mobility of ZnO films are enhanced at a higher temperature.

**Figure 7:**
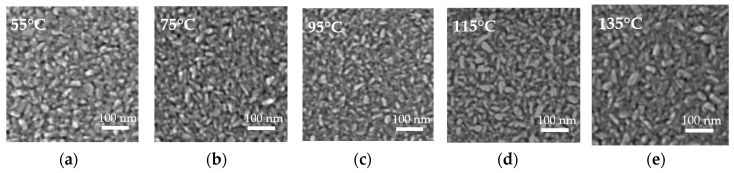
Field emission scanning electron microscopy (FESEM) images of sALD-ZnO films deposited at (**a**) 55 °C, (**b**) 75 °C, (**c**) 95 °C, (**d**) 115 °C, and (**e**) 135 °C.

Figure 7 shows the FESEM images of the ZnO films deposited on silicon wafers at different substrate temperatures. The surfaces of the films consist of rice-like grains [20,21,35]. The grains seem to grow larger as the substrate temperature increases slightly.

### 3.5. ZnO Film on PET Substrate

**Figure 8:**
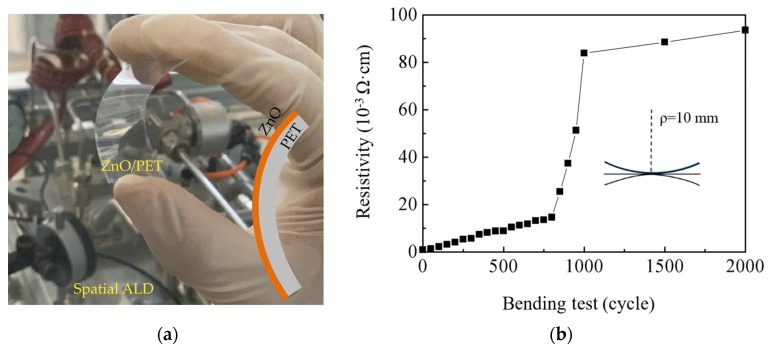
(**a**) A photograph of a bent ZnO/PET sample; (**b**) the bending test results.

Figure 8a shows a photograph of a bent ZnO/PET sample. Interestingly, the pristine ZnO film on the PET substrate exhibited a lower initial resistivity of 9.1 × 10^−4^ Ω·cm than that on the glass substrate. Figure 8b shows the bending test results. We tested five PET samples totally and similar results were obtained. The deflection apparently causes degradation in the film resistivity, which first gradually increases and then dramatically increases with the bending cycles. This variation trend of the resistivity is similar to those reported in the literature [16]. We speculate that the gradual increase in resistivity is possibly due to the defect’s creation by the deflection; possible cracking of the film may eventually happen as the defects accumulate to some extent and lead to a dramatic increase in resistivity, which is more likely caused by the tensile strain induced by downward bending rather than the compressive strain brought by upward bending. After being bent for 2000 cycles, the ZnO film still exhibits an acceptably low resistivity of 9.4 × 10^−2^ Ω·cm, which indicates excellent bending stability of the film.

## 4. Conclusions

ZnO thin films were prepared by spatial ALD with DEZ and H_2_O as precursors at different substrate temperatures. The presented sALD process shows a higher deposition rate than conventional ALD, and is hence of special interest for industrial application due to a possibly higher throughput. The transmittance and refractive indexes of ZnO films are rarely affected by the deposition temperature. A low resistivity of (3.2–9.0) × 10^−3^ Ω·cm was obtained and it only slightly decreased with the deposition temperature, possibly due to the crystal development. Therefore, this process makes it possible to deposit a high-quality ZnO film at a temperature as low as 75 °C, and hence, it can be applied to highly temperature-sensitive, flexible substrates. Finally, a ZnO film deposited on a PET substrate exhibits high electrical reliability after the bending test. This technique provides a wide process window for depositing ZnO thin films and can meet the requirements of low-temperature deposition in flexible electronics.

## Figures and Tables

**Table 1:** sALD-ZnO deposition parameters.

Parameter	Value
Substrate temperature (°C)	55–135
Substrate holder move speed (cm/s)	15
Distance between spray and sub. (mm)	0.3
H_2_O carry gas flow rate (sccm)	400
H_2_O dilute gas flow rate (sccm)	800
DEZ carry gas flow rate (sccm)	100
DEZ dilute gas flow rate (sccm)	3000
Precursor concentration (cc/cm^3^)	40
